# Non-participation in breast screening in Denmark: Sociodemographic determinants

**DOI:** 10.1186/s12889-024-19547-x

**Published:** 2024-07-29

**Authors:** My von Euler-Chelpin, George Napolitano, Elsebeth Lynge, Søren Borstrøm, Ilse Vejborg

**Affiliations:** 1 Department of Public Health, University of Copenhagen, Copenhagen K, 1014 Denmark; 2 Zealand University Hospital, Nykøbing Falster, Denmark; 3 Department of Breast Imaging/Capital Mammography Screening Program, Copenhagen University Hospital Herlev, Gentofte, Denmark

**Keywords:** Screening, Breast cancer, Mammography, Participation, Epidemiology

## Abstract

**Background:**

Internationally, non-participation in breast screening increased with decreasing level of education indicating importance of information campaigns to enhance awareness of screening. However, in Denmark in the 1990s the association between education and non-participation was U-shaped. We therefore analyzed recent Danish data.

**Methods:**

Data derived from the Capital Region of Denmark, biennial, organized breast screening program 2008–2020, where women aged 50–69 were personally invited to screening. Non-participation was measured as number of women with no participation out of women eligible for at least three invitations. Sociodemographic determinants were identified by linkage to public registers. Results were reported as age adjusted odds ratios (OR) of non-participation including 95% confidence intervals (CI).

**Results:**

Among 196,085 women, 86% participated. Using women with low education as baseline, the OR for professional bachelors was 0.64; and for academics 0.75. The strongest determinants of non-participation were being non-married OR 2.03; born outside Denmark OR 2.04; being self-employed OR 1.67; retired OR 3.12; on public support OR 3.66; or having co-morbidity OR 1.56.

**Conclusion:**

The U-shaped association between education and non-participation in breast screening prevailed. The data further indicated that screening participation was low in women with pertinent health and social problems.

**Supplementary Information:**

The online version contains supplementary material available at 10.1186/s12889-024-19547-x.

## Background

Breast cancer is the most common cancer in women [1]. Screening for breast cancer with mammography has been implemented in many countries, with the aim of reducing breast cancer mortality by early treatment [2–4]. However, for organized screening programs to be effective, high coverage is necessary. The European Union initiative to tackle the breast cancer burden includes that 90% of the EU population who qualify for breast cancer screening is offered screening by 2025 [5].

In a recent systematic review of studies published between 2010 and 2021, non-participation in breast screening programs was found to be associated with low income, younger age, low education, living distantly from the screening unit, being unmarried, being an immigrant, and having a male family physician [6].

In Denmark, breast screening started in the capital of Copenhagen in 1991 and in the county of Fyn in 1993. During the first four biennial invitation rounds of these programs, a u-shaped association was found between level of education and non-participation in the programs. In Copenhagen, using secretaries/sales assistants as baseline, the age adjusted relative risk (RR) of non-participation was 1.65 (95% confidence interval (CI) 1.37–1.99) in academics and 1.60 (95% CI 1.48–1.73) in women with only primary education [7]. A similar pattern was found for the Fyn program. In these early programs, the pattern of non-participation by level of education thus differed from the inverse association reported in the international literature.

Around 2000, Denmark experienced an intensive debate about the benefit and harms of breast screening [8]. This debate may have affected the attitude of well-educated women towards screening. It is interesting to see if the pattern of non-participation changed to be more in line with the international trend after the screening debate subsided. Nationwide breast screening started in Denmark in 2007/8 organized by the five regions, and by end of 2010 all women in the target age group had been invited. In the present study, we investigated the association between level of education and other socioeconomic factors and non-participation in breast screening in the Capital Region of Denmark in the time-period 2008 to 2020.

## Materials and methods

The Capital Region (RegionH) with Copenhagen and neighboring areas includes nearly one-third of the Danish population. The roll-out of breast screening in the entire RegionH took place in 2008–2009 [9]. At the implementation of the program, all women aged 50–69 years were personally invited if they lived in the region at the time when women with their respective birth-month were invited. Women were re-invited in the next invitation round, provided they still lived in the region, were 50–69 years, were not in treatment/control for breast cancer (which would normally last 18 months after the diagnosis of breast cancer), had no mammography within the last 6 months, and had not asked the screening center to be exempted from invitation. Women turning 50 and women aged 50–69 moving to RegionH were invited to screening according to their birth-month. From 2018 onwards, women with previous breast cancer were furthermore invited up to the age of 79 years. Time and screening location were indicated in the invitation letter, but changeable by web- or telephone contact to the screening center. Non-responding women were sent one reminder. The screening program was organized in invitation rounds of approximately two years, Supplementary Table 1.

For the present study we retrieved data from 2008 to 2020 from the RegionH mammography screening database. For each woman, the data retrieved included the unique Danish personal identity number, date of birth, invitation dates, and participation defined as a mammography after an invitation date and before the next invitation date or two years.

Some women included in this dataset will have been close to the upper end of screening age in 2008, while other women will recently have entered the dataset when they turned 50 years. Depending on their age at entry, the women will therefore have been eligible for screening between 1 and 6 times. Furthermore, after one invitation a woman could inform the screening center that she did not want further invitation. In order to take these two limitations into account, we restricted the analysis to women who had been eligible for at least three consecutive invitation rounds. Our analysis therefore covered women who turned 50 years before 17 November 2016 (end of fourth invitation round) and who were not older than 62 years on 9 January 2008 (start of first invitation round). A woman in this population was defined as participant in screening if she had at least one participation registered during the period 2008–2020, and as non-participant if she had no participation registered. To elucidate the possible impact of varying number of screenings offered, we made a sensitivity analysis including only women aged 50–56 at time of recruitment and all offered screening 6 times.

The women were stratified by age 50–54, 55–59, and 60–62 at first invitation after start of the RegionH program. Sociodemographic data defined by status at the date of first invitation were retrieved from registers in Statistics Denmark. Marital status was divided into married/living in registered partnership or not, including divorced, separated, widowed, or single. Birthplace as being in Denmark or elsewhere. Occupation was divided into “self-employed persons”, “employees”, “retired persons”, and “others” including other economically inactive women.

Education was highest achieved education. The classification of education was based on the Danish version of ISCED2011, see Supplementary Tables 2, and it was divided into “low education” (ISCED 10 + 20) including for instance unskilled workers and assistant nurses; “short term education” (ISCED 30 + 40) including short sales and administration education; “professional bachelor education” (ISCED 50 + 60) including nurses and teachers ; “academics” (ISCED 70 + 80) including persons with at least a master´s university education; and “missing” (ISCED 90) including mostly immigrants with no education registered in Denmark. Data on hospital contacts including diseases in the Charlson co-morbidity index; for codes see Supplementary Table 3, were retrieved for the period 2003–2020 and classified into 0 or 1 + contact within five years prior to first invitation.

Crude and age-adjusted ORs for non-participation vs. participation by independent variables were calculated with 95% confidence intervals (CIs). SAS 9.4 was used for statistical analyses, Copyright (c) 2016 by SAS Institute Inc., Cary, NC, USA.

Approval by Faculty of Health and Medical Sciences, University of Copenhagen, Ref. no.: 514 − 0238/18-3000, served as ethical clearance. According to Danish legislation, informed consent is not required for register-based studies without contact to patients, relatives and/or treating physicians.

## Results

The entire dataset included 319,136 women, of whom 196,085 had experienced at least three consecutive invitations rounds. Among the studied 196,085 women, 167,764 women had at least one participation registered, equal to 86%, Table 1. Participation rates at 89% were reached by married women; by employees; and by professional bachelors. Participation rates at or below 80% were found for non-married women; for women with at least one registered co-morbidity; for women born outside Denmark; for retired women; for women on other support; and for the small group of women with no education registered in Denmark.

**Table 1:** Women present during at least three consecutive invitation rounds to breast screening in Capital Region (RegionH), Denmark, 2008–2020

	Participants	Non-participants	Total	% Participants
Study population	167,764	28,321	196,085	85.6
Age*				
50–54	108,099	18,678	126,777	85.3
55–59	42,287	6709	48,996	86.3
60–62	17,378	2934	20,312	85.6
Marital status				
Married/cohabitation	107,124	13,172	120,296	89.1
Other	60,640	15,149	75,789	80.0
Birthplace				
Denmark	148,055	22,270	170,325	86.9
Other	19,709	6051	25,760	76.5
Occupation				
Self-employed	8500	1713	10,213	83.2
Employee	131,523	15,764	147,287	89.3
Retired	16,271	5793	22,064	73.7
Other	11,470	5051	16,521	69.4
Education				
Low	96,413	18,070	114,483	84.2
Short term	7771	1047	8818	88.1
Professional bachelors	42,415	5126	47,541	89.2
Academic	18,454	2606	21,060	87.6
Missing	2711	1472	4183	64.8
Co-morbidity				
None	151,053	24,185	175,238	86.2
One+	16,711	4136	20,847	80.2

* age at first invitation after start of the screening program

The co-morbidities associated with particularly low participation rates were aids 59%, dementia 61%, hemiplegia 62%, and liver diseases 64%, Table 2. Women previously diagnosed with breast cancer also had low participation at 73%. The largest groups of women with co-morbidities were those with vascular and pulmonary diseases, and both of these groups had participation rates at 82%.

**Table 2:** Women present during at least three consecutive invitations rounds to breast screening in Capital Region (RegionH), Denmark, 2008–2020, by specific diseases in the Charlson co-morbidity index

	Participants	Non-participants	Total	% Participants
Total	167,764	28,321	196,085	85.6
No disease	151,053	24,185	175,238	86.2
At least one disease	16,711	4136	20,847	80.2
Any disease*				
Dementia	46	29	75	61.3
Vascular disease	3499	760	4259	82.2
Pulmonary Disease (CPD)	3444	753	4197	82.1
Connective Tissue Disease	1509	238	1747	86.4
Ulcerous Disease	587	169	756	77.6
Liver Disease	544	307	851	63.9
Diabetes	2700	653	3353	80.5
Renal Diseases	422	110	532	79.3
Hemiplegia	68	41	109	62.4
AIDS	89	61	150	59.3
Cancer (excl Breast Cancer)**	2822	791	3613	78.1
Breast Cancer IBC + DCIS	2539	947	3486	72.8

*A given woman can have more than one disease. In total 23,128 diseases were registered, equal to an average of 1.11 diseases per sick woman

** Cancer (excl Breast Cancer), but including non-melanoma skin cancer

**Fig. 1:**
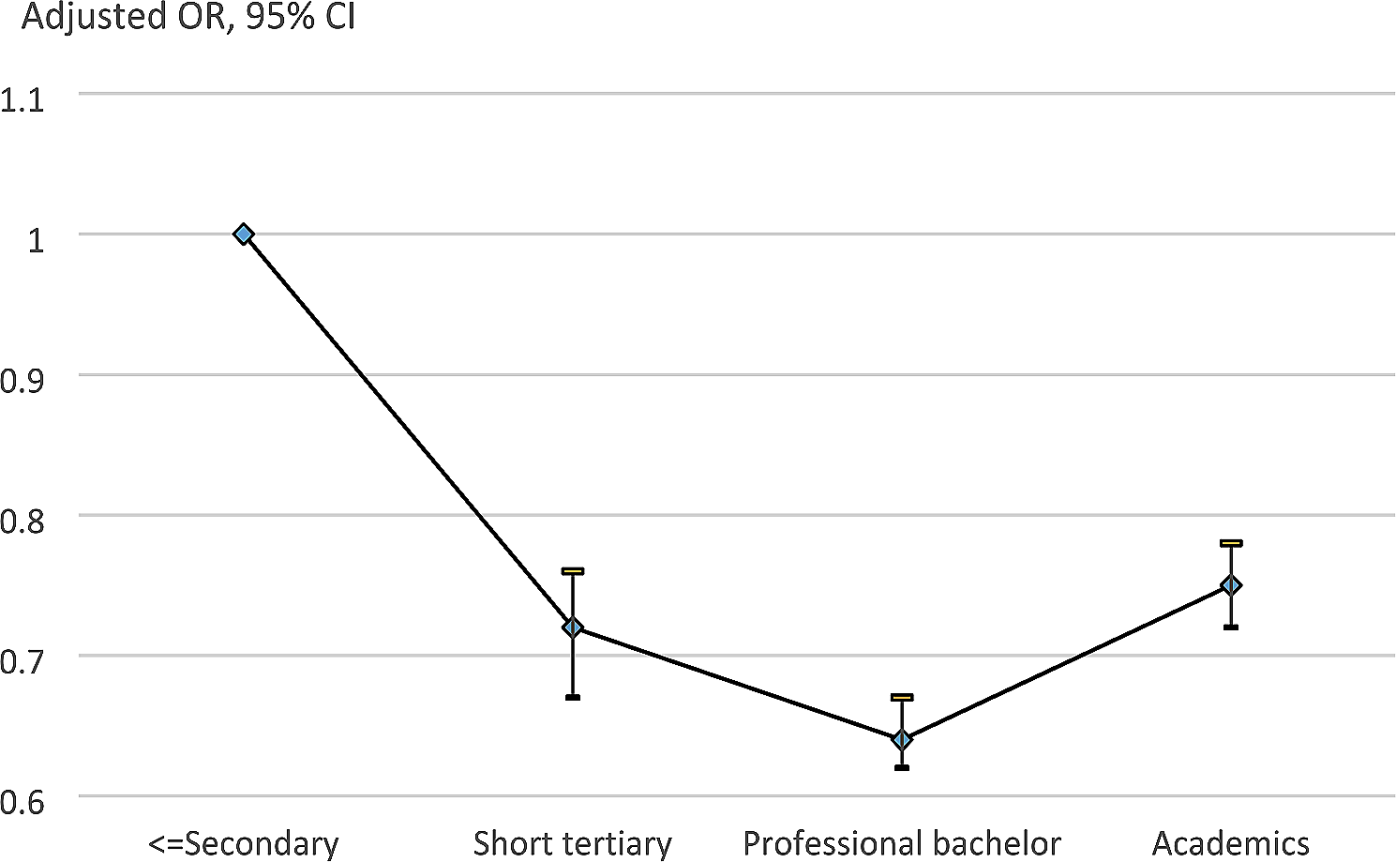
Odds of non-participation in breast screening by education, Capital Region (Region H), Denmark, 2008–2020

The OR for non-participation in screening was 0.92 (95% CI 0.89–0.95) for women aged 55–59 and 0.98 (95% CI 0.94–1.02) for those aged 60–62 using women aged 50–54 at first invitation as baseline, Table 3. For non-married women vs. married women, the adjusted OR was 2.03 (95% CI 1.98–2.08). For women born outside vs. those born inside Denmark, the adjusted OR was 2.04 (95% CI 1.97–2.10). Using employees as baseline, self-employed women had an adjusted OR for non-participation of 1.67 (95% CI 1.58–1.77); retired women of 3.12 (95% CI 3.02–3.24); and women on other support of 3.66 (95% CI 3.53–3.80). Using women with low education as baseline, women with short term education had an adjusted OR of 0.72 (95% CI 0.67–0.76); professional bachelors of 0.64 (95% CI 0.62–0.67); academics of 0.75 (95% CI 0.72–0.78); and women with missing information on education of 2.88 (95% CI 2.70–3.07), Fig. 1. In the sensitivity analysis of women aged 50–56 at time of recruitment, the ORs by level of education were for short term education 0.62; for professional bachelor 0.62; for academic 0.73; and for missing education 2.75, thus closely following the pattern for the entire cohort. Women with at least one co-morbidity as compared to those without had an adjusted OR of non-participation of 1.56 (95% CI 1.50–1.61).

**Table 3:** Odds ratios (OR) and 95% confidence interval (CI) for non-participation vs. participation in breast screening, Capital Region (RegionH), Denmark, 2008–2020

	Crude		Age-adjusted	
Age*	OR	95% CI	OR	95% CI
50–54	1		1	
55–59	0.92	0.89–0.95	-	-
60–62	0.98	0.94–1.02	-	-
Marital status				
Married/cohabitation	1	-	1	-
Other	2.03	1.98–2.08	2.03	1.98–2.08
Birth place				
Denmark	1	-	1	-
Other	2.04	1.98–2.11	2.04	1.97–2.10
Occupation				
Self-employed	1.68	1.59–1.78	1.67	1.58–1.77
Employee	1	-	1	-
Retired	2.97	2.87–3.07	3.12	3.02–3.24
Other	3.67	3.54–3.81	3.66	3.53–3.80
Education				
Low	1	.-	1	-
Short term	0.72	0.67–0.77	0.72	0.67–0.76
Professional bachelors	0.64	0.62–0.67	0.64	0.62–0.67
Academic	0.75	0.72–0.79	0.75	0.72–0.78
Missing	2.90	2.71–3.09	2.88	2.70–3.07
Co-morbidity				
None	1	-	1	-
One+	1.55	1.49–1.60	1.56	1.50–1.61

*age at first invitation after start of the screening program

## Discussion

### Main results

Among Danish women aged 50–69 years and covered by the RegionH organized breast screening program for at least three consecutive invitation rounds, only 14% had not participated at least once. The strongest predictor of non-participation was other health or social problems illustrated by more than doubled ORs for non-participation among retired women and other women on public support, and an elevated OR also for women hospitalized for co-morbidities. Foreigners also had a relatively low participation illustrated by elevated ORs for non-participation for women born outside Denmark and/or with no education registered in Denmark. Independent predictors of non-participation were also being non-married or being a self-employed woman.

Those aged 50–54 at first invitation, included many who turned 50 during the study period of 2008–2020, had higher non-participation than those already aged 55–62 at the first invitation in 2008–2009. Concerning education, using women with only low education as baseline, all other women had a lower level of non-participation. But non-participation did not decrease with increasing level of education; the OR for non-participation was 0.64 for professional bachelors while 0.75 for academics with at least a master´s degree, and there was no overlap between the 95% CIs.

### Previous studies

In the first invitation round to breast screening in the Central Denmark Region in 2008–2009, one in five invited women did not participate, and non-participants were almost equally divided between women who actively cancelled their appointment and women who passively did not show up on the booking date [10]. The highest proportions of non-participants were found for immigrants, women with low household income, living alone, with co-morbidities, and low education. In another study of basically the same population, the highest adjusted non-participation prevalence ratios (PR) were found for women on early pension, on unemployment benefit or being self-employed; being non-western immigrant; being single; with low household income; with > 60 km to the screening site; and without access to a vehicle [11]. It was noteworthy that in the adjusted analysis only minor differences were seen between women by length of education; using 11–15 years as baseline; the PR was 1.10 (95% CI 1.08–1.13) for women with ≤10 years, and 1.15 (95% CI 1.11–1.18) for women with 15 + years of education [11]. Non-participation increased by number of co-morbidities both within the previous two years and before [12], and a similar pattern was seen in Danish national data from 2007 to 2010 [13].

When Health Survey data from 2006 on social support were linked with breast screening data from the Central Denmark Region for 2008–2009, low social support was found to be associated with non-participation in screening [14]. For instance, rarely or never spending time with persons living elsewhere; adjusted PR 1.69 (1.26–2.26); or not having someone to talk to about personal concerns, adjusted PR 1.42 (95% CI 1.14–1.77). In the same data, u-shaped association were found between both physical and mental self-assessed health and perceived stress and non-participation in breast screening; associations that remained after control for presence of chronic diseases [15]. When Health Survey data from 2017 on health literacy were linked with breast screening data from the Central Denmark Region for 2016–2017, only a weak, statistically non-significant association was found between low health literacy and non-participation in screening [16].

A targeted investigation of all women in Denmark diagnosed with intellectual disabilities showed a more than five-fold OR for non-participation in breast screening in the patients compared with age-matched controls [17]. Breast cancer survivors are known to have a lifelong increased risk of recurrent disease and from 2018 onwards they were invited to screening up to the age of 79 years. However, another national Danish investigation showed breast cancer survivors to have lower screening participation than other women during the first 6 years following the breast cancer diagnosis [18].

When Ding et al. reviewed the available literature on determinants of non-participation in breast screening, they included studies published between 2010 and 2021 [6]. In the meta-analysis, they found strong risk estimates for non-participation for immigrants, OR 2.64 (95% CI 2.48–2.82); for unmarried women OR 1.68 (95% CI 1.32–2.14), and for having a male family physician OR 1.43 (95% CI 1.20–1.61). Although statistically significant ORs were found also for low income, young age, low education, and distance to screening unit, all of these estimates were of moderate size. For low vs. high education, the OR was 1.18 (95% CI 1.05–1.32), and as commented on by the authors this effect of low education on non-participation was considerably smaller than the OR of 1.61 (95% CI 1.36–1.91) reported in a previous meta-analysis including studies published between 2000 and 2013 [19]. This could point to a diminishing impact in recent years of educational level on non-participation in breast screening. It should be noted though that the Ding et al. meta-analysis data could not be seen as independent evidence of the studies from Denmark, as eight of the 29 studies reviewed by Ding et al. were from Denmark.

### Strengths and limitations

It was a strength of our study that we used population-based registers of high quality in terms of coverage and accuracy. It was furthermore a strength that we analyzed a large dataset resulting in narrow confidence intervals for the risk estimates. It was a limitation that we had no individual follow-up of women for moving out of the RegionH or death, but movements out of the area was limited by the fact that RegionH constituted nearly one third of the Danish population, and the probability of death at the upper age-limit of 70 years was below 1.5% [20].

Unfortunately, the classification of highest achieved education used by Statistics Denmark had changed since we analyzed data from 1991 to 1999 from the first two and smaller breast screening programs in Denmark [7], so it was not possible to make a one-to-one comparison. We could not divide women on pension by type of pension. In Denmark in 2008 women aged 65 were entitled to old age pension; a limit that was from 2019 to 2021 gradually increased to age 67. Given that screening was offered up to the age of 70, women first invited at age 54–62 reached entitlement for old age pension during the study period. Retired women outside this group received early pension mostly due to illness. Women receiving other types of public support will also mostly have been ill or in other ways unable to work. It was a limitation that our co-morbidity classification was based only on data on hospital contacts, which means that for instance diabetes often treated in primary care will be underrepresented in the data.

We limited the study population to women under risk of at least three invitations to screening, but some women had more chances, as women aged 50–56 at first invitation could have had been invited six times. However, the sensitivity analysis showed results for women aged 50–56 at recruitment to be in accordance with the results for the entire cohort. Our data covered only the Capital Region of Denmark, but results were in line with observations from the first years of screening in the Central Denmark Region [10–12]. Finally, it was a limitation that we could study only sociodemographic determinants of non- participation in screening as the register data did not include information on lifestyle, health awareness, or emotional barriers to screening.

### Public health implications

Our results opened for various possibilities for improving participation in breast screening.

First, non-participation was high in women born/educated outside Denmark. It was possible that information about the screening program did not successfully reach these women. Second, non-participation was high in self-employed women such as shop owners and hairdressers, which could reflect that they had difficulties in leaving their jobs in the opening hours of the screening units. Third, non-participation was high in non-married women including single, divorced, and widowed women, and efforts are warranted to reach these women especially if they are nulliparous with an increased risk of breast cancer [21].

Fourth, and quantitatively most important, the highest ORs for non-participation were found for retired and other women on public support, and for women with co-morbidities. These observations were in line with results on breast screening from the Central Denmark Region. Furthermore, non-participants in breast screening have been found to have low participation in cervical screening [22], and in a recent Danish study the strongest determinants of non-participation in cervical screening were illness and being on early pension and other support [23].

Notably, non-participation in breast screening did not decrease with increasing level of education. One could speculate that it is not the years of education but whether you have some sort of education that counts, and that this is a proxy for healthy behavior. Another possibility is that some well-education women do deliberately not prioritize screening in their busy life. Although not representing fully independent evidence, comparison from meta-analyses pointed to a diminishing impact of education on screening participation. If this tendency proves to hold in other studies, lack of knowledge about the screening program does not anymore seem to be a main hindrance for participation in cancer screening. In the future, the traditional tool of information campaigns to enhance screening participation will probably not work in countries with organised screening programs and well-educated women. Instead, the challenge will be to find ways to reach women with pertinent health and social problems.

## Conclusion

In conclusion, about 15% of women in the Capital Region of Denmark offered organized breast screening did not participate, despite personal invitations followed by reminders. Traditionally, non-participation in screening has been associated with low education and consequently lack of information. The recent data from Denmark presented here indicated that non-participation did not decrease with increasing level of education. The data indicated that more likely other pertinent health and social problems and practical obstacles could explain non-participation in breast screening. In this case, general information campaigns will not increase screening participation but instead targeted interventions towards the non-participating groups are needed.

### Electronic supplementary material

Below is the link to the electronic supplementary material.

Supplementary Material 1

Supplementary Material 2

Supplementary Material 3

## Data Availability

The data that support the findings of this study are available from Statistics Denmark but restrictions apply to the availability of these data, which were used under license for the current study, and so are not publicly available. Data are however available from the authors upon reasonable request and with permission of Denmark Statistics.
