# Supplementary material for: Non-participation in breast screening in Denmark: Sociodemographic determinants
Source: BMC Public Health. 2024 Jul 29;24:2024. doi: 10.1186/s12889-024-19547-x (PMC11285456; doi:10.1186/s12889-024-19547-x)
Supplement: Supplementary file 1 — Supplementary Material 1 [file 12889_2024_19547_MOESM1_ESM.docx]

Supplementary Table 1. Invitation rounds in breast screening in Capital Region (RegionH), Denmark, 2008-2020

| Invitation round | Starting date | Ending date | Length in years |
| --- | --- | --- | --- |
| 1 | 20 December 2007 | 31 January 2010 | 2.1 |
| 2 | 31 January 2010 | 31 December 2011 | 2.0 |
| 3 | 1 January 2012 | 30 April 2014 | 2.3 |
| 4 | 1 May 2014 | 31 June 2016 | 2.2 |
| 5 | 1 July 2016 | 1 July 2018 | 2.0 |
| 6 | 1 July 2018 | 1 October 2020 | 2.3 |
