# Supplementary material for: Non-participation in breast screening in Denmark: Sociodemographic determinants
Source: BMC Public Health. 2024 Jul 29;24:2024. doi: 10.1186/s12889-024-19547-x (PMC11285456; doi:10.1186/s12889-024-19547-x)
Supplement: Supplementary file 2 — Supplementary Material 2 [file 12889_2024_19547_MOESM2_ESM.docx]

Supplementary Table 2. Classification of education.

| Our classification | Examples | DISCED-15 code | Text |
| --- | --- | --- | --- |
| Low | Unskilled workers,  Assistant nurses | 10 | Primary education only |
|  |  | 20 | Lower secondary education |
| Short term | Sales and administration | 30+40 | Upper secondary education |
| Professional bachelor | Nurses,  School teachers | 50 | Short tertiary |
|  |  | 60 | Bachelor |
| Academic | Higher education teachers, economists, lawyers, physicians, etc. | 70 | Master degree |
|  |  | 80 | PhD-degree |
| Missing | Mostly immigrants without an education finalized in Denmark | 90 | Unknown |
