# Supplementary material for: Non-participation in breast screening in Denmark: Sociodemographic determinants
Source: BMC Public Health. 2024 Jul 29;24:2024. doi: 10.1186/s12889-024-19547-x (PMC11285456; doi:10.1186/s12889-024-19547-x)
Supplement: Supplementary file 3 — Supplementary Material 3 [file 12889_2024_19547_MOESM3_ESM.docx]

Supplementary Table 3. Comorbidity Codes: ICD-10

| Myocardial Infarction | I21.x, I22.x, I25.x, |
| --- | --- |
| Congestive Heart Failure | I42.x, I43.x, I50.x, |
| Peripheral Vascular Disease | I70.x, I71.x, I73.x, I79.x, K55.x, Z95.x |
| Cerebrovascular Disease | G45.x, G46.x, I60.x-I69.x |
| Dementia | F00.x –F03.x, F05.x, G30.x |
| Chronic Pulmonary Disease and Pneumonia | I27.x, J40.x-J47.x, J60.x- J68.x, J70.x |
| Rheumatic Disease | M05.x, M06.x, M31.x-M36.x |
| Peptic Ulcer Disease | K25.x-K28.x |
| Mild Liver Disease | B18.x, K70.x K71.x, K73.x, K74.x, K76.x, Z94.x |
| Diabetes | E10.x-E14.x |
| Hemiplegia or paraplegia | G80.x-G84.x |
| Renal Disease | N03.x, N05.x, N18.x, N19.x, Z49.x |
| Malignancy | C00.x-C26.x, C30.x-C34.x, C37.x-C41.x, C43.x, C45.x-58.x, C60.x-C76.x, C81.x- C85.x, C88.x, C90.x-C97.x |
| Moderate or Severe Liver Disease | I85.x, K72.x |
| Metastatic Solid Tumor (excl. Breast cancer) | C77.x-C80.x (excl. C50) |
| Breast cancer | C50 |
| AIDS/HIV | B20.x-B22.x, B24.x |
